# Comparative Dentoskeletal Effects of Two Fixed Systems in Treating Class II Malocclusion: A Retrospective Cohort Study

**DOI:** 10.3390/healthcare14080989

**Published:** 2026-04-09

**Authors:** Mauro Lorusso, Michele Tepedino, Gianvittorio Ferritto, Elena D’Angelo, Fariba Esperouz, Lucio Lo Russo, Domenico Ciavarella

**Affiliations:** 1 Department of Clinical and Experimental Medicine, Dental School of Foggia, University of Foggia, 71122 Foggia, Italydomenico.ciavarella@unifg.it (D.C.); 2 Department of Biotechnological and Applied Clinical Sciences, University of L’Aquila, 67100 L’Aquila, Italy

**Keywords:** class II malocclusion, Carriere motion appliance, RME II system, functional appliances, growth modification

## Abstract

**Objectives:** Class II malocclusion is a frequent orthodontic problem in growing patients, and understanding the dentoskeletal effects of different treatment approaches is essential for selecting the most appropriate therapeutic strategy. This study aimed to compare the skeletal and dentoalveolar effects of the Carriere Motion appliance (CMA) and the Rapid Maxillary Expander II (RME II) system in growing patients with Class II malocclusion, using an untreated control group. **Methods**: This study included 86 growing patients with skeletal Class II Division 1 malocclusion, divided into three groups: RME II (n = 28), CMA (n = 28), and untreated controls (n = 30). Lateral cephalograms were obtained at baseline (T0) and after Class II correction (T1). Skeletal and dentoalveolar variables were assessed, and intergroup differences in treatment changes were analyzed using appropriate statistical tests with correction for multiple comparisons. **Results:** Both treatment groups showed significantly greater reductions in overjet than the control group, with no significant difference between the two appliances. The CMA group showed a greater reduction in overbite, whereas the RME II group showed greater reductions in the A point–Nasion–B point (ANB) angle and greater increases in mandibular length (Condylion–Gnathion; Co-Gn) compared with both the control and CMA groups. **Conclusions:** Both appliances were effective in correcting Class II malocclusion during growth. However, the CMA was mainly associated with dentoalveolar correction and vertical changes, whereas the RME II system induced more evident skeletal modifications.

## 1. Introduction

Class II malocclusion is one of the most prevalent sagittal discrepancies in orthodontic practice and represents a substantial proportion of treatment demand in both growing patients and adults. It is generally defined by a distal relationship of the mandible relative to the maxilla, reflecting an anteroposterior discrepancy of skeletal, dentoalveolar, or combined origin [1]. Epidemiological evidence indicates that Class II malocclusion is a common condition worldwide, underscoring its clinical relevance. The distribution of malocclusion varies across geographic regions, age groups, and ethnic backgrounds [2,3,4]. In the permanent dentition, Class I malocclusion is the most prevalent pattern (74.7%), followed by Class II (19.56%) and Class III (5.93%). A similar trend has been reported in the mixed dentition, where Class I accounts for 73% of cases, Class II for 23%, and Class III for 4%. Among the various ethnic groups, Caucasian populations show the highest reported prevalence of Class II malocclusion, both in the permanent (23%) and mixed dentition (26%) [2,3,4]. Malocclusion encompasses different sagittal patterns, conventionally classified as Class I, Class II, and Class III, each reflecting distinct dentoskeletal relationships and therapeutic implications. Although the present study specifically focuses on Class II malocclusion, this broader classification is clinically relevant because treatment strategies and growth-related responses differ substantially across malocclusion types.

Class II malocclusion has been reported to be associated with maxillary transverse deficiency, a condition that may predispose patients to maxillary canine impaction [5,6,7,8].

Because of its heterogeneous etiology and clinical presentation, treatment planning requires careful diagnosis and an individualized therapeutic approach [3].

In growing patients, early treatment of Class II malocclusion may take advantage of residual growth to improve sagittal relationships, although the effects of treatment are often a combination of skeletal and dentoalveolar changes [9,10,11].

In non-growing patients, treatment options typically include orthodontic camouflage with intermaxillary elastics, distalization mechanics, or skeletal anchorage systems, while orthognathic surgery remains the treatment of choice for severe skeletal discrepancies [12,13,14,15,16]. In recent years, technological advances, such as temporary anchorage devices and aligner-based mandibular advancement protocols, have expanded the therapeutic possibilities for Class II correction across different age groups [17]. Given the heterogeneity of etiological factors and growth patterns, contemporary management of Class II malocclusion requires an evidence-based, patient-centered approach that integrates accurate diagnosis, appropriate timing, and biomechanical efficiency to achieve stable functional and esthetic outcomes [18].

The therapeutic management of Class II malocclusion in growing patients is complex, as in addition to appropriate treatment timing, patient compliance is fundamental for successful outcomes, particularly in adhering to the prescribed therapy and the proper use of appliances or auxiliaries such as intermaxillary elastics.

The Rapid Maxillary Expander II (RME II) System is a fixed functional appliance designed for the treatment of skeletal Class II malocclusion associated with maxillary transverse deficiency. The device combines a Hyrax-type maxillary expander with a lower lingual arch and intermaxillary Class II elastics, allowing simultaneous correction of transverse and sagittal discrepancies during growth [19].

The Carriere Motion appliance (CMA, Henry Schein Orthodontics, Melville, NY, USA) is a fixed sagittal distalization system designed for the correction of Class II malocclusion during the initial phase of orthodontic treatment. The appliance consists of a rigid bar extending from the maxillary canine to the first molar, combined with Class II intermaxillary elastics anchored to the mandibular dentition, typically stabilized with a lower lingual arch or rigid retainer [20]. Its primary objective is to distalize the maxillary buccal segment as a unit, correcting the sagittal discrepancy before comprehensive orthodontic alignment [21].

Although these appliances operate through different biomechanical mechanisms, their effectiveness largely depends on patient compliance with intermaxillary elastic wear. To date, no studies have directly compared the effects of these two appliances. Therefore, the objective of the present study was to evaluate and compare the skeletal and dentoalveolar effects produced by these two devices relative to an untreated control group.

The null hypothesis of the present study was that, in growing patients with Class II malocclusion, no significant differences would be observed in the dentoskeletal effects produced by the Carriere Motion appliance and the Rapid Maxillary Expander II system.

## 2. Materials and Methods

Participants were enrolled according to the following inclusion criteria: bilateral full Class II molar relationships, an overjet greater than 5 mm, skeletal Class II Division 1 malocclusion characterized by mandibular retrusion as determined by cephalometric analysis, chronological age between 11 and 13 years, and a skeletal maturation stage corresponding to CS2–CS3 based on the cervical vertebral maturation method. Exclusion criteria included the presence of periodontal disease, orofacial inflammatory conditions, dental agenesis, congenital syndromes, previous orthodontic therapy, and temporomandibular joint disorders. An overjet > 5 mm was adopted as an inclusion criterion because this threshold is considered clinically relevant in patients with increased sagittal discrepancy and is associated with an increased risk of traumatic injury to the anterior teeth [22].

This investigation was conducted following the Strengthening the Reporting of Observational Studies in Epidemiology (STROBE) recommendations (Supplementary Materials: STROBE checklist) [23]. All procedures described in the study protocol were consistent with the principles of the Declaration of Helsinki and were approved by the Ethics Committee. Patient records were collected and analyzed retrospectively under conditions of complete anonymity, and written informed consent was obtained from the participants’ parents.

A priori sample size calculation was performed using G*Power software (version 3.1.9.2; Franz Faul, University of Kiel, Kiel, Germany). The analysis indicated that at least 28 subjects per group were necessary to detect a large effect size of 0.4 [24] with a one-way analysis of variance (ANOVA), assuming a significance level (α) of 0.05 and a statistical power (1 − β) of 0.90.

The sample included three groups: one group received treatment with the RME II system, one group was treated with the Carriere Motion appliance, and one group consisted of untreated control subjects. Groups treated with the RME II system and the Carriere Motion appliance were retrospectively identified among patients treated at the Department of Orthodontics, University of Foggia, through a chronological selection of records from March 2021 to June 2024.

All lateral cephalograms of the treated groups were obtained using standardized radiographic procedures routinely adopted at the study center, with patients positioned in natural head posture and teeth in maximum intercuspation. The untreated control group was obtained from the historical Michigan Growth Study. Since the control group was derived from a historical U.S. sample, whereas the treated group consisted of patients treated at an Italian university setting, possible differences in population characteristics, ethnicity, growth patterns, and radiographic protocols should be considered when interpreting the comparisons. Nevertheless, the same cephalometric variables and reference landmarks were applied to all groups to allow comparative analysis.

Treatment was considered complete upon achievement of bilateral Class I molar and canine relationships. All participants were instructed to wear the appliance for approximately 16 h per day throughout the active treatment phase. The subjects in control group were selected from the American Association of Orthodontists Foundation Craniofacial Growth Legacy Collection (Michigan Growth Study; https://www.aaoflegacycollection.org).

### 2.1. GROUP RME II System

The group included 28 patients (14 males and 14 females) with a mean age of 11.8 years (SD = 0.5), all treated with the RME II System. The mean treatment duration was 12 months.

**Figure 1:**
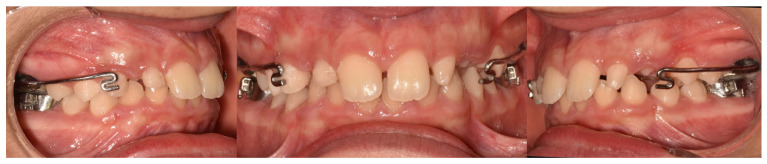
The RME II System appliance.

The RME II System is a functional orthopedic appliance that incorporates a Hyrax-type expander anchored to permanent first molars. The design includes two rigid buccal arms extending anteriorly toward the canines, along with a lower lingual arch equipped with hooks for the attachment of intermaxillary elastics. Representative clinical images of the appliance are presented in Figure 1.

The expansion protocol involved adjustments at 21-day intervals to achieve gradual transverse development. Patients were instructed to wear Class II elastics for approximately 16 h per day throughout the active phase. The elastic routine consisted of 4.5 oz, 3/8-inch elastics for four months, followed by 6-oz, 3/8-inch elastics for one month, and finally 4-oz, 3/8-inch elastics for two months.

### 2.2. Carriere Motion Appliance GROUP

**Figure 2:**
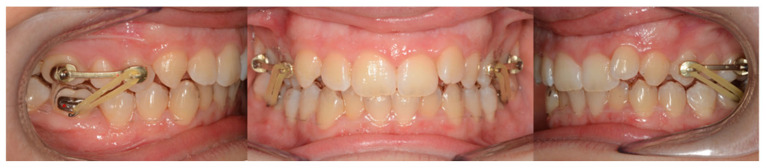
A Carriere Motion appliance extending from the first premolar to the first molar.

The group comprised 28 patients (13 male and 15 female) with a mean age of 11.5 years (SD = 0.7 years) treated with Carriere Motion appliance (Figure 2).

The CMA is a fixed device designed to correct Class II malocclusion. It consists of a rigid, anatomically contoured bar extending from the maxillary canine or first premolar to the maxillary first molar on each side. The appliance is bonded to the canine and molar using preformed pads adapted to the tooth surfaces, allowing the maxillary buccal segment to be moved as a single unit during distalization. Its shape is preadjusted to follow the curvature of the maxillary arch, minimizing unwanted rotations and enabling controlled distal movement of the posterior segment.

The CMA was fitted in accordance with the manufacturer’s guidelines. In the mandibular arch, buccal tubes equipped with elastic hooks were bonded to the first molars, and a transparent retainer with a thickness of 1 mm was inserted.

Elastic wear involved Force 1™ elastics (1/4-inch, 6 oz) and Force 2™ elastics (3/16-inch, 8 oz; Henry Schein Orthodontics, Savannah, GA, USA), which were worn throughout the CMA phase of treatment. After this stage, full fixed appliances were installed using preadjusted 0.022-inch MBT brackets.

Class correction was achieved through the use of intermaxillary elastics attached from the distal end of the maxillary appliance to hooks on the mandibular molars in conjunction with rigid retainer. The elastic protocol involves full-time wear (approximately 20–22 h per day), with progressive changes in force levels according to the manufacturer’s instructions and the patient’s response to treatment.

#### 2.2.1. Control Group

This group consisted of 30 untreated patients (16 males and 14 females) with a mean age of 11 years (SD = 0.4). These subjects were sampled from the Michigan Medical Library and were matched to the other groups based on age and sex to ensure comparability.

#### 2.2.2. Cephalometric Analysis

All measurements were obtained by a single operator on digitized cephalograms using a digital caliper (Screen Caliper, version 4.0). The cephalometric analysis was carried out by the same examiner, who had extensive training in computerized cephalometric evaluation, using Dolphin Imaging software (version 11.0; Chatsworth, CA, USA).

**Figure 3:**
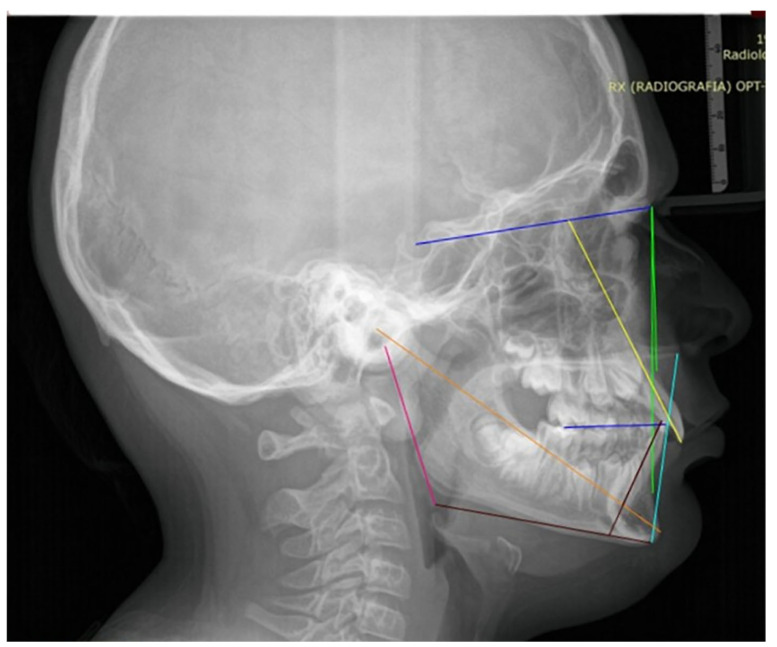
Cephalometric tracing illustrating the variables included in the analysis.

The measurements were performed under blinded conditions: the orthodontist responsible for the analyses was not involved in patient treatment, and all records were anonymized prior to evaluation. The following skeletal and dental cephalometric variables were assessed: SN–MP, ANB, lower facial height (LFH), Co–Gn, U1–SN, IMPA, overjet, and overbite. The variables used are shown in Figure 3.

### 2.3. Statistical Analysis

All cephalometric tracings and measurements were performed by a single examiner to ensure methodological consistency and to minimize inter-examiner variability. Intra-examiner reliability was assessed by repeated measurements, and the method error was calculated using Dahlberg’s formula.

To reduce random error, all cephalometric and dental measurements were recorded twice. The magnitude of random error for each variable was calculated using Dahlberg’s formula (S = ∑d^2^/2N), where d represents the difference between the first and second measurements and N indicates the number of radiographs analyzed [25]. The estimated random error ranged from 0.18 to 0.29 mm for linear measurements and from 0.27° to 0.38° for angular measurements.

Data distribution was assessed using the Shapiro–Wilk test. Within-group comparisons between T0 (pre-treatment) and T1 (post-treatment) were performed using either paired *t*-tests or the Wilcoxon test, depending on data normality (Table 1). Intergroup differences were analyzed by means of one-way analysis of variance (ANOVA) on the T1–T0 changes for each variable (Table 2), followed by Tukey’s post hoc test (Table 3). In cases where variance homogeneity was not met, Welch’s ANOVA was applied, followed by the Games-Howell post hoc test. To reduce the risk of Type I error, the Holm–Bonferroni correction was applied to both intra-group and intergroup comparisons. The level of statistical significance was set at *p* < 0.05.

## 3. Results

After Holm–Bonferroni correction, both the Carriere and RME II system groups showed significant changes from T0 to T1 (Table 1).

In the Carriere group, treatment led to a significant improvement in sagittal skeletal relationship, with the ANB angle decreasing from 5.61 ± 1.65° at T0 to 3.88 ± 1.37° at T1 (Holm-adjusted *p* = 0.006). A significant reduction was also observed in overjet (from 5.34 ± 1.97 mm to 4.00 ± 0.81 mm, Holm *p* = 0.010) and overbite (from 3.32 ± 1.45 mm to 2.57 ± 0.97 mm, Holm *p* = 0.048). In addition, significant increases were found in lower facial height (LFH, ANS-Me), which rose from 54.17 ± 2.57 mm to 56.22 ± 3.03 mm (Holm *p* = 0.007), and in mandibular length (Co-Gn), which increased from 102.61 ± 4.16 mm to 104.48 ± 3.63 mm (Holm *p* = 0.008). No significant changes were found for SN-GoMe, L1-GoMe, or 1-SN.

In the RME II system group, a similar pattern was observed. The ANB angle significantly decreased from 5.85 ± 2.16° at T0 to 3.96 ± 1.93° at T1 (*p* = 0.008). Significant reductions were also found in overjet (from 7.74 ± 2.17 mm to 3.81 ± 1.12 mm, *p* = 0.008) and overbite (from 2.74 ± 1.95 mm to 1.86 ± 1.03 mm, *p* = 0.030). Moreover, L1-GoMe increased significantly from 93.77 ± 8.10° to 97.49 ± 8.46° (*p* = 0.008). Vertical changes were also observed, with LFH increasing from 54.94 ± 4.99 mm to 57.81 ± 5.32 mm (*p* = 0.008) and Co-Gn from 100.70 ± 6.41 mm to 105.10 ± 6.31 mm (*p* = 0.008). No significant changes were found for SN-GoMe or 1-SN after correction.

Since Levene’s test showed heterogeneity of variances for several variables, Welch ANOVA was used when appropriate. After Holm–Bonferroni correction, significant intergroup differences were found for SN-GoMe, LFH, Co-Gn, overbite, overjet, and ANB, whereas no significant differences were found for IMPA or 1 + SN.

For SN-GoMe, all groups differed significantly from each other. The RME II group showed lower values than both the control group (−1.82°, *p* = 0.002) and the Carriere group (−3.94°, *p* < 0.001), whereas the Carriere group showed higher values than the control group (2.12°, *p* = 0.022). For LFH and Co-Gn, both treatment groups showed significantly greater increases than the control group, and significant differences were also found between the RME II and Carriere groups. No significant intergroup differences were observed for 1 + SN or IMPA.

Regarding dental variables, the Carriere group showed a significantly greater reduction in overbite than both the control and RME II groups (*p* < 0.002). For overjet, both treatment groups showed significantly greater reductions than the control group (*p* < 0.001), with no significant difference between the two treatment groups. Finally, for ANB, the RME II group showed a significantly greater reduction than both the control and Carriere groups (*p* < 0.001), whereas no significant difference was found between the Carriere and control groups.

## 4. Discussion

The present study compared the dentoskeletal effects of two fixed Class II treatment approaches, the Carriere Motion appliance and the Rapid Maxillary Expander II (RME II) system, in growing patients with Class II malocclusion, using an untreated control group for comparison. Both appliances produced significant improvements in sagittal correction; however, the magnitude and pattern of the observed changes differed, suggesting distinct mechanisms of action. Accordingly, the null hypothesis was rejected. Overall, the findings indicate that the Carriere Motion appliance acted predominantly through dentoalveolar effects, whereas the RME II system showed a greater skeletal contribution. Although both protocols resulted in clinically relevant improvements in sagittal relationship, the biological pathways underlying these changes were not equivalent. This distinction is clinically relevant, as it suggests that treatment selection should consider not only the sagittal discrepancy itself but also the patient’s skeletal and vertical pattern, transverse relationships, growth potential, and overall treatment objectives. The Carriere Motion appliance, originally introduced by Luis Carrière in 2004 as the Carriere Distalizer, is based on the “Sagittal First” philosophy, according to which correction of the sagittal discrepancy is achieved before comprehensive orthodontic alignment, with the aim of simplifying the subsequent treatment phases [26].

In the Carriere group, a significant reduction in ANB angle and overjet was observed, indicating an improvement in sagittal discrepancy. These findings are consistent with previous reports showing that the Carriere Motion appliance is effective in Class II correction mainly through dentoalveolar mechanisms, including distal movement or distal tipping of the maxillary posterior segment and mesial movement of the mandibular dentition [27,28]. This interpretation is also supported by Elsaharty et al. [29], who reported that the Carriere Motion appliance primarily produces dentoalveolar correction, whereas clinically relevant skeletal effects appear to be limited

In the present study, the Carriere group also showed a significant increase in lower facial height and mandibular length, together with a tendency toward increased divergence. In contrast, Wilson et al. [30] and Kim-Berman et al. [27] did not report significant changes in divergence after treatment with the Carriere Motion appliance. This discrepancy may be related to differences in sample characteristics, growth stage, observation period, or anchorage design. In the present sample, the vertical changes observed in the Carriere group may reflect both the side effects of intermaxillary elastics and the contribution of residual craniofacial growth.

Although lower incisor proclination and upper incisor retroclination were observed in the Carriere group, these changes did not remain statistically significant after Holm–Bonferroni correction. Nevertheless, this pattern is in agreement with the dentoalveolar effects commonly described for Class II elastics and fixed Class II correction protocols [31,32], suggesting that dental compensation contributed, at least in part, to sagittal correction in this group.

In the RME II group, significant reductions in ANB, overjet, and overbite were also observed. These findings are in agreement with previous studies [14,19], in which improvement in sagittal relationship was mainly attributed to mandibular advancement and growth, as reflected by the significant increase in Co-Gn. In this group, the reduction in overjet appears to have resulted from a combination of skeletal and dentoalveolar effects. On the one hand, maxillary expansion may promote a more favorable mandibular posture and facilitate sagittal correction during the mixed dentition phase [33]; on the other hand, the observed increase in mandibular length suggests that residual mandibular growth also contributed substantially to the treatment effect. Similar to the Carriere group, lower incisor proclination and upper incisor retroclination were observed in the RME II group, further supporting the role of dentoalveolar adaptation in overjet correction. However, unlike the Carriere protocol, the RME II system was associated with a more evident skeletal effect, particularly in terms of mandibular length increase and ANB reduction.

The intergroup analysis further supports this interpretation. After correction for multiple comparisons, significant differences among groups were observed in both sagittal and vertical variables, confirming the effectiveness of both appliances relative to the untreated controls. However, the comparison between the two treatment protocols is particularly informative. The Carriere group differed from controls mainly in dental and vertical variables, whereas the RME II group showed additional differences in mandibular length and sagittal skeletal relationship, supporting a stronger orthopedic effect. In particular, the greater reduction in ANB and the larger increase in Co-Gn observed in the RME II group support a more pronounced skeletal contribution, whereas the greater reduction in overbite and the vertical changes observed in the Carriere group are consistent with the known effects of intermaxillary elastics. Similarly, the greater lower incisor proclination observed in the Carriere group is in agreement with previous evidence [21,34], again suggesting that dentoalveolar compensation played a more relevant role in this protocol. Taken together, these findings indicate that the Carriere appliance may be associated with a greater vertical component, whereas the RME II protocol may provide more balanced skeletal changes.

From a clinical perspective, both the Carriere Motion appliance and the RME II system may be considered effective options for the treatment of Class II malocclusion in growing patients. However, the two appliances differed in the pattern of correction achieved. The Carriere appliance appeared to act primarily through dentoalveolar changes, with a greater vertical component, whereas the RME II system showed more pronounced skeletal effects, particularly in relation to ANB reduction and mandibular length increase. Accordingly, appliance selection should not be based solely on the sagittal discrepancy, but should also consider the patient’s skeletal pattern, transverse relationships, growth stage, and specific treatment objectives. Particular attention should be given to neuromuscular evaluation and force balance analysis, as these factors may facilitate the early detection of functional alterations potentially affecting craniofacial growth and treatment response. Early identification of such imbalances may support timely intervention with functional appliances aimed at restoring muscular equilibrium and promoting more favorable skeletal development.

The present findings should be interpreted with caution in light of several limitations. The retrospective design may have introduced selection bias and reduced control over potential confounding variables. The relatively short observation period precludes any assessment of the long-term stability of the observed changes. Moreover, treatment effects could not be fully disentangled from physiological growth, particularly with respect to mandibular length. Although the groups were matched for age and sex, slight differences in mean age may still have affected growth-related outcomes. The use of a historical untreated control group should also be considered a limitation, as differences in sample characteristics and radiographic protocols may have affected comparability. Although adherence to elastic wear was routinely evaluated during monthly follow-up visits, compliance may still have represented a confounding factor, given that both treatment protocols relied on intermaxillary elastics despite differing in biomechanics.

Therefore, further studies with larger and more homogeneous samples, contemporaneous well-matched control groups, longer follow-up, and more comprehensive functional assessments are needed to confirm the present findings and clarify their long-term stability.

## 5. Conclusions

Within the limitations of this study, it can be concluded that both the Carriere Motion appliance and the RME II system proved effective in correcting Class II Division 1 malocclusion in growing patients but with different actions.

The selection of the appliance should be based on a comprehensive evaluation of the patient’s skeletal pattern, residual growth potential, and compliance in order to achieve a more individualized and biologically appropriate correction.

## Figures and Tables

**Table 1:** Paired *t*-test or Wilcoxon test comparing variables at T0 and T1 in the Carriere group. Paired *t*-test or Wilcoxon test comparing variables at T0 and T1 in the RME II system group.

Variable	Group	Mean T0	SD T0	Median T0	Mean T1	SD T1	Median T1	Shapiro–Wilk (ΔT0 − T1)	Test	*p*	Holm–Bonferroni *p*
ANB	Carriere	5.61	1.65	5.80	3.88	1.37	3.90	0.290	*t*-test	<0.001	**0.006**
SN-GoMe	Carriere	32.52	5.83	33.00	34.22	5.11	33.00	0.836	*t*-test	0.025	0.050
L1-GoMe	Carriere	93.19	7.16	92.20	96.45	4.75	96.20	0.982	*t*-test	0.017	0.051
1-SN	Carriere	112.87	3.08	113.00	110.22	8.03	111.00	0.689	*t*-test	0.151	0.151
Overjet	Carriere	5.34	1.97	4.90	4.00	0.81	4.00	0.516	*t*-test	0.002	**0.010**
Overbite	Carriere	3.32	1.45	3.20	2.57	0.97	2.70	0.081	*t*-test	0.012	**0.048**
LFH (ANS-Me)	Carriere	54.17	2.57	54.00	56.22	3.03	55.00	0.015	Wilcoxon	0.001	**0.007**
Co-Gn	Carriere	102.61	4.16	101.00	104.48	3.63	103.00	0.025	Wilcoxon	0.001	**0.008**
ANB	RME II	5.85	2.16	6.20	3.96	1.93	4.70	0.019	Wilcoxon	<0.001	**0.008**
SN-GoMe	RME II	32.93	4.25	33.90	33.08	4.61	32.20	0.002	Wilcoxon	0.230	0.230
L1-GoMe	RME II	93.77	8.10	94.10	97.49	8.46	97.90	0.044	Wilcoxon	<0.001	**0.008**
1-SN	RME II	106.21	5.66	105.20	103.74	6.05	104.00	0.089	*t*-test	0.038	0.076
Overjet	RME II	7.74	2.17	7.60	3.81	1.12	3.70	0.055	*t*-test	<0.001	**0.008**
Overbite	RME II	2.74	1.95	3.00	1.86	1.03	1.70	0.001	Wilcoxon	0.010	**0.030**
LFH	RME II	54.94	4.99	55.90	57.81	5.32	58.90	0.222	*t*-test	<0.001	**0.008**
Co-Gn	RME II	100.70	6.41	101.30	105.10	6.31	104.40	0.011	Wilcoxon	<0.001	**0.008**

**Table 2:** Intergroup comparison using one-way ANOVA or Welch ANOVA according to Levene’s test for homogeneity of variances.

Variable	Levene	Test	F	df1	df2	*p*	Holm–Bonferroni *p*	Effect Size (η^2^)
SN-Go-Me	0.002	Welch	12.158	2	42.503	0.001	**0.008**	0.364
LFH	0.006	Welch	13.666	2	51.692	0.001	**0.007**	0.346
CO-GN	0.004	Welch	35.164	2	50.014	0.001	**0.006**	0.584
1 + SN	0.071	ANOVA	2.110	2	80	0.128	0.128	0.050
IMPA	0.037	Welch	3.015	2	47.904	0.058	0.116	0.112
Overbite	0.040	Welch	17.325	2	53.132	0.001	**0.005**	0.395
Overjet	0.145	ANOVA	20.688	2	80	0.001	**0.004**	0.341
ANB	0.472	ANOVA	18.649	2	80	0.001	**0.003**	0.318

**Table 3:** Post hoc comparisons among the groups using Tukey or Games–Howell tests.

Variable	Comparison	Post Hoc Test	Mean Difference	Std. Error	95% CI Lower	95% CI Upper	*p*-Value
SN-Go-Me	RME II vs. Control	Games-Howell	−1.815	0.505	−3.040	−0.590	0.002 *
	Carriere vs. Control		2.121	0.747	0.269	3.972	0.022 *
	RME II vs. Carriere		−3.936	0.835	−5.971	−1.900	<0.001 **
LFH	RME II vs. Control	Games-Howell	−5.467	1.041	−7.975	−2.959	<0.001 **
	Carriere vs. Control		−3.314	0.928	−5.569	−1.059	0.003 *
	RME II vs. Carriere		−2.153	0.747	−3.960	−0.347	0.016 *
CO-GN	RME II vs. Control	Games-Howell	−4.335	1.294	−7.458	−1.212	0.004 *
	Carriere vs. Control		−8.655	1.145	−11.449	−5.862	<0.001 **
	RME II vs. Carriere		4.320	0.849	2.261	6.379	<0.001 **
1 + SN	RME II vs. Control	Tukey	−3.250	1.957	−7.924	1.424	0.227
	Carriere vs. Control		−3.872	2.101	−8.889	1.145	0.162
	RME II vs. Carriere		−0.622	2.101	−5.639	4.395	0.953
IMPA	RME II vs. Control	Games-Howell	−0.661	1.091	−3.286	1.964	0.817
	Carriere vs. Control		2.926	1.507	−0.745	6.596	0.141
	RME II vs. Carriere		−3.587	1.456	−7.146	−0.027	0.048 *
Overbite	RME II vs. Control	Games-Howell	−1.110	0.549	−2.430	0.210	0.116
	Carriere vs. Control		−2.758	0.489	−3.940	−1.575	<0.001 **
	RME II vs. Carriere		−1.648	0.460	−2.758	−0.537	0.002 *
Overjet	RME II vs. Control	Tukey	−3.590	0.567	−4.944	−2.236	<0.001 **
	Carriere vs. Control		−2.403	0.609	−3.857	−0.950	<0.001 **
	RME II vs. Carriere		−1.187	0.609	−2.640	0.267	0.132
ANB	RME II vs. Control	Tukey	−2.150	0.357	−3.003	−1.297	<0.001 **
	Carriere vs. Control		−0.720	0.384	−1.636	0.195	0.152
	RME II vs. Carriere		−1.430	0.384	−2.345	−0.514	0.001 **

* *p* < 0.05, ** *p* < 0.001.

## Data Availability

The data presented in this study are available upon request from the corresponding author due to privacy reasons.

## Supplementary Materials

The following supporting information can be downloaded at: https://www.mdpi.com/article/10.3390/healthcare14080989/s1, STROBE checklist.
